# [Retracted] Liriodenine induces the apoptosis of human laryngocarcinoma cells via the upregulation of p53 expression

**DOI:** 10.3892/ol.2025.14972

**Published:** 2025-03-10

**Authors:** Liang Li, Ying Xu, Binquan Wang

Oncol Lett 9: 1121–1127, 2015; DOI: 10.3892/ol.2014.2834

Following the publication of this paper, it was drawn to the Editor's attention by a concerned reader that the same data panel had apparently been featured in Fig. 3B on p. 1125 to show the results from differently performed experiments, and that the western blotting data shown in Fig. 1E on p. 1123 had subsequently appeared at a later stage in a different research article written by different authors at different research institutes. This prompted the Editorial Office to perform its own independent analysis of the data in the paper, and this revealed that TUNEL assay data in Fig. 1C and certain of the aforementioned western blotting data were strikingly similar to data appearing in different form in other articles written by different authors at different research institutes that had either already been published elsewhere prior to the submission of this paper to *Oncology Letters*, or were under consideration for publication at around the same time. In view of the fact that certain of the abovementioned data had already apparently been published previously, the Editor of *Oncology Letters* has decided that this paper should be retracted from the Journal. The authors were asked for an explanation to account for these concerns, but the Editorial Office did not receive a satisfactory reply. The Editor apologizes to the readership for any inconvenience caused.

